# Proactive Telephone Smoking Cessation Counseling Tailored to Parents: Results of a Randomized Controlled Effectiveness Trial

**DOI:** 10.3390/ijerph16152730

**Published:** 2019-07-31

**Authors:** Tessa Scheffers-van Schayck, Roy Otten, Rutger C.M.E. Engels, Marloes Kleinjan

**Affiliations:** 1 Trimbos Institute, Netherlands Institute of Mental Health and Addiction, P.O. Box 725, 3500 AS Utrecht, The Netherlands; 2 Department of Interdisciplinary Social Sciences, Utrecht University, P.O. Box 80140, 3508 TC Utrecht, The Netherlands; 3 Research and Development, Pluryn, P.O. Box 53, 6500 AB Nijmegen, The Netherlands; 4 ASU REACH Institute, Department of Psychology, Arizona State University, P.O. Box 876005, Tempe, AZ 85287-6005, USA; 5 Developmental Psychopathology, Radboud University, P.O. Box 9104, 6500 HE Nijmegen, The Netherlands; 6 Executive Board, Erasmus University Rotterdam, P.O. 1738, 3000 DR Rotterdam, The Netherlands

**Keywords:** smoking cessation, parents, telephone counseling, effectiveness trial

## Abstract

A recent Dutch efficacy trial showed the efficacy of a telephone smoking cessation counseling tailored to smoking parents. Currently, it is unknown whether such telephone counseling would be effective under more real-world conditions. This study aimed to examine the effectiveness of parent-tailored telephone smoking cessation counseling in a two-arm randomized controlled effectiveness trial and whether the effectiveness depended on the recruitment approaches that were used to recruit parents (mass media vs. health care). In total, 87 parents received either telephone counseling (intervention) or a self-help brochure (control). Parents were asked to complete questionnaires at baseline and three months post-intervention. Results showed that the odds of reporting 7-day point-prevalence abstinence at three months post-intervention was 7.54 higher for parents who received telephone counseling than for parents in the control condition (53.3% vs. 13.2%, 95% CI = 2.49–22.84). Because inclusion was lower than anticipated, interaction-effects of condition and recruitment approach could not be interpreted. The present study demonstrates that the parent-tailored smoking cessation telephone counseling is effective in helping parents to quit smoking. Yet, before large-scale implementation, future research should focus on how recruitment of parents via the recruitment approaches could be improved.

## 1. Introduction

Worldwide, children are currently more heavily exposed to secondhand smoke (SHS) than any other age group [1]. Children’s exposure to SHS predominantly occurs at home where their close relatives (e.g., parents) smoke [1], making it difficult for children in such situations to avoid or protect themselves from the hazards of SHS. There is ample evidence that children’s exposure to SHS can lead to serious short- and long-term health consequences [2,3,4]. Parental smoking cessation is the most effective way to avoid harm to children from exposure to SHS at home [5]. Moreover, parental smoking cessation reduces the likelihood that children will start smoking themselves when they are older [6]. 

To date, several effective smoking cessation interventions for parents have been developed and examined [5,7,8,9,10,11,12,13,14]. Recently, a telephone smoking cessation counseling intervention tailored to parents was examined in the Netherlands [15]. Parents who smoked (*N* = 512) were recruited through primary schools in a two-arm randomized controlled trial (RCT). Parents received telephone counseling (intervention condition) or a self-help brochure (control condition). Results revealed that at three months post-intervention, the odds of reporting 7-day point-prevalence (PPA) was 6.89 higher for parents in the intervention condition compared to parents in the control condition (95% CI = 4.18–11.36). Among parents who did not quit smoking at three months post-intervention, parents in the intervention condition smoked fewer cigarettes per day, made more attempts to quit, and more often showed 24 h abstinence compared to parents in the control condition (*p* < 0.001). Even in the long term, at twelve months post-intervention, more parents who received telephone counseling showed abstinence compared to parents in the control condition. In short, this telephone counseling proved to be effective in helping parents to quit smoking, thereby protecting children from exposure to their parents’ smoking.

Although, the results of Schuck et al. (2014) [15] are promising, and telephone counseling certainly has the potential to decrease the number of parents that smoke, the results are limited in that the counseling was examined in an efficacy trial. This means that the telephone counseling was tested under optimal controlled circumstances (e.g., parents received 100 euros for participating in the study and the telephone counseling was offered for free, whereas often smokers have to pay for smoking cessation counseling in the Netherlands (depending on the type of health insurance they have)) [16]. Thus, it remains unclear how the telephone counseling would function under more real-world circumstances and whether the findings of the efficacy trial are generalizable to everyday practice. This is an important limitation to address, as interventions worthy of implementation need to be effective under conditions that resemble the conditions in the real world [17].

In order to establish the optimal conditions under which an intervention can be most successfully implemented, it is also necessary to know whether the effectiveness of an intervention depends on the recruitment approach that was used. For example, perhaps telephone counseling tailored to parents is more effective when parents are recruited by a pediatrician as compared to social media. Thus far, many studies have examined the effectiveness of smoking cessation interventions for parents in (youth) health care settings [5,12,13,14,18,19,20,21]. A few studies recruited smoking parents through primary schools [15,22], a birth cohort study [7], or via other channels (e.g., flyers and cultural events) [10]. To our knowledge, however, no studies have explored whether the effectiveness of a smoking cessation intervention for parents depends on the recruitment approach that was used. However, a profound understanding of the effectiveness of the telephone counseling intervention and the role of recruitment is paramount prior to widespread implementation.

In order to gain more insight into the potential impact of the telephone smoking cessation counseling that was previously examined in the aforementioned efficacy trial [15], the present study had two aims. The first aim was to examine the effectiveness of the telephone counseling in an two-arm effectiveness RCT. The second aim was to test whether the effectiveness of the telephone counseling depended on the manner of recruitment (health care vs. mass media). Based on Schuck and colleagues (2014) [15], we hypothesized that parents who received telephone counseling (intervention condition) would be more likely to report 7-day PPA at three months post-intervention compared to parents who received a self-help brochure (control condition). In addition, we hypothesized that telephone counseling would be more effective for parents who were recruited via the health care approach than for parents who were recruited via the mass media approach. The rationale behind this hypothesis was that these parents could be more motivated to quit smoking, since health care professionals addressed their smoking and its consequences for their children’s health. Moreover, discussing smoking cessation with health care professionals could be a ‘teachable moment’ for parents [20,23].

## 2. Materials and Methods 

### 2.1. Study Design

The effectiveness of the telephone smoking cessation counseling was examined in a two-arm single-blind RCT, which was part of a larger study in which the implementation of the telephone counseling was also assessed by adopting an effectiveness-implementation hybrid design [24,25]. The present study only reports the results of the RCT, as the results of the implementation study will be reported elsewhere. Parents who smoke were randomly assigned to the intervention condition (telephone counseling) or to the control condition (a self-help brochure). The study was registered in the Netherlands Trial Register (NTR6092), and the ethics committee of the Trimbos Institute approved the study’s protocol (201607_52-1606). More information on the design, recruitment, data collection, and treatment conditions of this study can be found in the study protocol [26]. No important changes to methods were made after the study protocol was published. 

### 2.2. Recruitment 

Parents who smoke were recruited in the Netherlands between September 2016 and September 2018. A mass media approach (i.e., primary schools and online mass media) and a health care approach (i.e., general health care and youth health care) were used for implementation and recruitment of parents. 

#### 2.2.1. Mass Media Approach

(1) Primary Schools

Between September and November 2016, 619 primary schools were randomly approached to distribute information letters among parents of children (aged 4–12 years). In total, 101 (16.3%) primary schools agreed to distribute invitation letters. The invitation letters were targeted to parents in general and were not personalized. The invitation letters included a description of the study’s aim and inclusion criteria, as well as the frequency of the assessments. In addition, parents were told that several family excursions would be raffled among the participating parents. The aim of the raffle was to encourage parents to complete the assessments, not to encourage parents to enroll in the study. To minimize performance bias, parents were told that the study aimed to examine the effectiveness of two smoking cessation interventions. Parents could register on the study website or by returning the form via mail (active informed consent). After registration, parents were called by professional smoking cessation counselors from SineFuma (one of the Dutch certified quit lines), and additional information was provided (e.g., parents who were randomized to the telephone counseling may have had to pay for the telephone counseling, depending on their health insurance). Parents could either confirm or withdraw their registration. Because fewer parents registered for the study than expected, the same 101 primary schools were approached again between October 2017 and January 2018 and were asked to distribute the information letter by e-mail or mail for a second time. In total, 77.0% of the primary schools agreed to do so.

(2) Online Mass Media

Parents were recruited through social media and two smoking cessation websites, including the Dutch national smoking cessation website (www.ikstopnu.nl [English translation: www.IQuitNow.nl]. Multiple paid Facebook and Instagram advertisements were created and deployed between September 2017 and May 2018. The Facebook advertisements and information on the websites aimed to recruit parents that were interested in smoking cessation. Parents who were interested in receiving a free, informal, and proactive phone call with a smoking cessation counselor could complete a short registration form (including name, telephone number, and e-mail address) on the website of SineFuma. Parents were contacted by a smoking cessation counselor within one week. During this phone call, parents were screened as to whether they could participate in the RCT by checking the eligibility criteria (see “participants” for more information). If parents met the inclusion criteria, more information about the RCT was provided. Parents who agreed to participate received an invitation letter by mail to confirm their participation and were asked to return the completed form by mail or on the study’s website. Telephone counseling was offered outside the study context to parents who did not meet the inclusion criteria or who did not want to participate in the study. 

#### 2.2.2. Health Care Approach

General health care and youth health care professionals (hereinafter health care professionals) were recruited via several methods, including visiting health care centers and conferences, giving presentations, mailings, social media, and word of mouth. Health care professionals who wanted to participate in the study could register by filling out a short registration form on a website from the Trimbos Institute that provides health care professionals with information about smoking. After registration, health care professionals were called by the research team and received more information about the study. 

In collaboration with a group of representatives of health care professionals, the research team developed a convenient and time-saving tool that they could use to refer parents to effective smoking cessation support (i.e., participating in the RCT or receiving telephone counseling). Health care professionals could register parents who were interested in smoking cessation via our website, telephone or fax. Subsequently, within one week, parents were called by a smoking cessation counselor from SineFuma. During this phone call, parents that met the inclusion criteria were asked to participate in our study. Parents who did not meet the inclusion criteria or who did not want to participate in the present study could receive the telephone counseling outside the study context. The registration process for the study was identical to the registration process through online mass media. Health care professionals could refer parents to our RCT or the telephone counseling between December 2016 and September 2018. 

### 2.3. Sample

To participate in the RCT, parents had to: (1) be a parent/caretaker of a child between 0 and 18 years old; (2) be at least a weekly smoker; (3) have the intention to quit smoking currently or in the future; and (4) give informed consent to participate. Pregnant women were excluded from the study, but telephone counseling was offered to them outside the study context. Based on the results of Schuck et al. [15], a power calculation (Stata 12.1, StataCorp., College Station, TX, USA) revealed that a sample size of 72 parents per recruitment approach (36 intervention and 36 control) was sufficient to detect 7-day PPA at the three months follow-up assessment with a large effect (p1 = 0.445, p2 = 0.121, power = 0.80, α = 0.05). Because we aimed at examining differences in smoking cessation rates between the two recruitment approaches, we doubled the target sample size from 72 to 144 parents. Thus, in anticipation of 10% drop-out, we aimed to recruit a minimum of 158 participating parents in total. However, due to lower inclusion rates than expected, a total of 83 smoking parents were recruited, randomized, and included in the analyses (42.2% male, M = 39.2, SD = 7.20). This means that we were still able to test our main hypothesis, but our hypothesis regarding the interaction effect of the recruitment approaches could only be tested in an exploratory manner. 

### 2.4. Data Collection

Parents were asked to fill out two questionnaires: a baseline assessment and a 3-month follow-up assessment. The web survey software application Jambo Research Software (2017) was used to collect parents’ answers. Parents received a personal invitation by e-mail to fill out two questionnaires at a secure website. After completion of the baseline assessment, parents were randomly assigned to one of the two trial conditions (i.e., telephone counseling condition vs. self-help brochure condition) by means of a computer algorithm with a block size of 2. True random codes were used from www.random.org. Stratified randomization was used based on educational level (i.e., low: no high school diploma/no vocational training; medium: vocational training or high school diploma; high: college degree), recruitment channel (i.e., primary schools, health care, youth health care, and online mass media), and number of cigarettes smoked per day (i.e., less than 10; 10–20; 21 or more). When parents lived in the same household and both participated (*n* = 3 parent couples), randomization was carried out at household level to avoid contamination. Randomization was concealed until parents were assigned to one of the two conditions and an automatic e-mail with the result was sent to the research team. 

Three months after parents had received the telephone counseling or self-help brochure, parents were approached to fill out the follow-up questionnaire online. A subsample of parents who reported that they had abstained from smoking for the previous seven days at the follow-up assessment were approached for biochemical validation. Home visits were conducted to collect saliva samples (NicAlert dipstick (Nyomax, Hasbrouck Heights, NJ, USA)). Parents who scored > 10 ng/mL were considered “smokers”.

### 2.5. Treatment Conditions

To minimize performance bias, parents were informed that the study aimed to examine the effectiveness of two smoking cessation interventions. Parents were blind to which of the two smoking cessation interventions was the intervention condition. The research team and the smoking cessation counselor that provided the telephone counseling were not blind to this information. 

#### 2.5.1. Telephone Counseling (Intervention Condition)

Parents who were allocated to the intervention condition received telephone counseling. The telephone counseling intervention included six proactive 20 min counseling phone calls over a period of 10 weeks. The counseling sessions were performed by one professional smoking cessation counselor of SineFuma who was thoroughly trained, experienced, and certified in delivering smoking cessation counseling. The counseling was based on Motivational Interviewing, which is an effective smoking cessation counseling approach [27,28]. Multiple topics were discussed during the counseling, including use of NRT, smoking history, withdrawal symptoms, cravings, and relapse prevention. The counselor followed a protocol on which topics to discuss during the sessions. However, the intensity and the order in which the topics were discussed could differ based on the needs and preferences of parents (tailoring). In addition to the telephone counseling, parents received a supplementary brochure, which was developed for the study. This tailor-made brochure (‘Rookvrije Ouders’ (English translation: ‘Smoke-free Parents’)) provided didactic information about smoking, smoking cessation, motivational messages, exercises, and tips that were relevant for parents who wanted to quit smoking. To guarantee quality and comprehensibility, professional counselors of SineFuma and communication experts of the Trimbos Institute were involved in the development of the brochure. Parents received the brochure at the start of the telephone counseling. 

#### 2.5.2. Self-help Brochure (Control Condition)

Parents who were assigned to the control condition received a self-help brochure by mail within one week after they had completed the baseline assessment. The brochure (‘Wat je zou moeten weten over stoppen met roken’ (English translation: ‘What you should know about smoking cessation’) was a 16-page, color-printed booklet developed by the Trimbos Institute. It included elements that have been shown to be effective (e.g., focusing on advantages of smoking cessation) and discussed several topics (e.g., consequences of smoking and available smoking cessation methods). At the end of the study, telephone counseling was offered to parents who were allocated to the control condition during the study. 

### 2.6. Measurements

#### 2.6.1. Primary Outcome

The primary outcome was 7-day PPA at three months post-intervention. At the follow-up assessment, parents were asked whether they had smoked (even a single puff) or used any other form of tobacco during the past seven days (yes/no). If parents answered “yes” on one or both questions they were considered “smokers”. 

#### 2.6.2. Secondary Outcomes

The secondary outcomes included: (1) occurrence of 24 h PPA at three months post-intervention (yes/no); (2) 14-day PPA at four weeks after the designated quit date (yes/no; Russel Standard (2005) [29]); and (3) use of nicotine replacement therapy (NRT; e.g., nicotine patches) or any smoking cessation medication (e.g., Champix; yes/no)). Among parents who did not report abstinence at three months post-intervention, the following secondary outcomes were tested: (1) increase in motivation to quit smoking during the study; (2) number of quit attempts during the study; (3) duration of quit attempts during the study (less than one week/one week or more); and (4) implementation of smoking restrictions at home (yes/no). With respect to the latter, parents were asked what rules they had concerning smoking in their house and could select from (a) “smoking is never allowed in the house”, (b) “smoking is sometimes allowed”, (c) “smoking is allowed in some rooms only”, and (d) “there are no rules about smoking in the house”. In line with Hyland et al. (2009) [30], parents who selected that smoking was never allowed in the house were considered to have a smoke-free home. A dichotomous scale was used with 0 = ‘not having a smoke-free home’ and 1 = ‘having a smoke-free home’. The outcomes ‘increase in motivation to quit during the study’ and ‘implementation of smoking restrictions at home’ were measured at both assessments. All other outcomes were only assessed at the three months follow-up assessment. No changes to the outcomes were made after the study protocol had been published [26]. 

### 2.7. Statistical Analyses

Data were analyzed according to the intent-to-treat (ITT) principle. Analyses were carried out using Statistical Package for the Social Sciences (SPSS), version 25 (IBM, Armonk, NY, USA). Parents who did not complete the follow-up assessment were considered “smokers” for the three smoking cessation outcomes (i.e., 7-day and 24 h PPA at three months post-intervention and 14-day PPA at four weeks after the designated quit date) [15]. In line with the efficacy trial [15], multiple imputation was used to handle missing data for additional outcomes. If significant correlations between baseline variables and primary and secondary outcomes were found, the baseline variables were included in the imputation model. Twenty imputed data sets were generated. Four parents were excluded from analyses, because they were pregnant (*n* = 2) or because they resigned from the study because they had been assigned to the control condition but needed to receive the telephone counseling (e.g., for medical reasons, *n* = 2).

Bivariate logistic regression models were used to compare cessation rates between the two conditions. Odds ratios (OR) were not adjusted for baseline characteristics, since none of the baseline characteristics were significantly correlated with the primary outcome. Independent t-tests were carried out for continuous outcomes, and chi-square tests were used for categorical outcomes. To determine the robustness of the primary outcome, a sensitivity analysis was carried out by considering parents who did not complete the follow-up assessment to be “quitters”. The interaction effect between condition and recruitment approach was tested in an exploratory manner by computing a product term and including this term in the logistic regression analysis. Because this study was underpowered to examine the interaction effect, additional logistic regression analyses were performed to gain more insight into the relationship between the recruitment approaches and the smoking cessation outcomes. These additional analyses are not reported in the trial registration and the study’s protocol [26]. Descriptive statistics (% for categorical variables and means and standard errors for continuous variables), ORs, 95% confidence intervals (CI), *χ*^2^, and *p*-values are reported. 

## 3. Results

**Figure 1:**
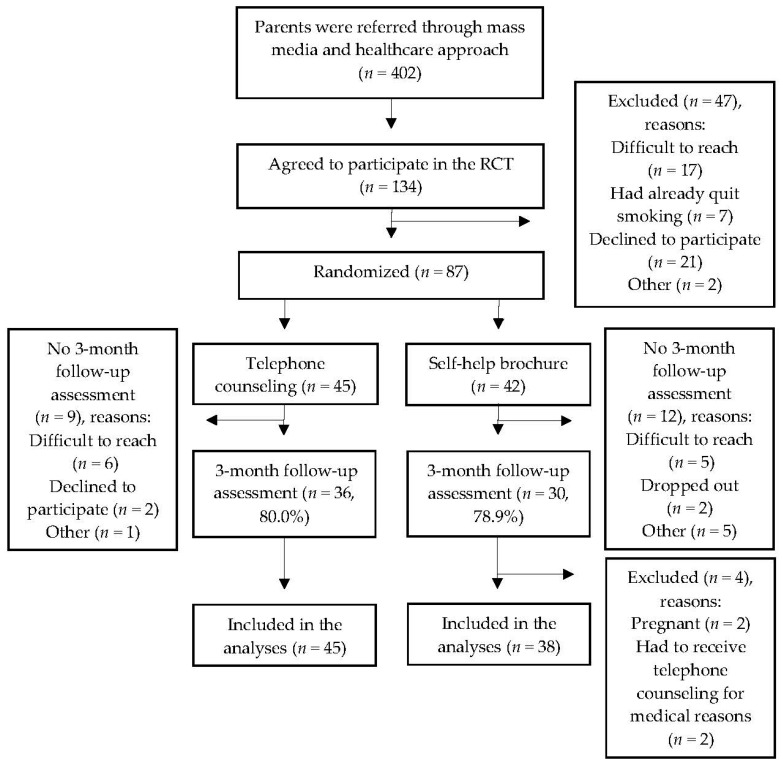
Flowchart of recruitment and data collection.

The flow of the participants, follow-up rates, and number analyzed are depicted in Figure 1. In total, 402 parents were recruited via the health care and mass media approach. Of these, 87 parents completed the baseline assessment and were randomized, and 83 parents were included in the analyses. The follow-up rate for the primary outcome was 79.5% (66 parents). Parents who did not complete the follow-up assessment were contacted by phone and e-mail, but most did not respond after these repeated requests. No significant difference was found in the follow-up rate between the intervention and control conditions (80.0% vs. 78.9%, *χ*^2^ = 0.01, *p* > 0.05). Parents lost at follow-up were more likely to use medical treatment and to be unemployed and were less likely to complete a house smoking ban compared to the remaining parents. 

### 3.1. Characteristics of the Participants

Table 1 presents the characteristics of the parents who were included in the analyses (*N* = 83). The mean age was 39.2 years and 42.2% of the parents were male. On average, parents had smoked 20.4 years and smoked 15.5 cigarettes per day. The majority of the parents (83.1%) reported that they had implemented a complete smoking ban at home. In total, 34.9% of the parents had a partner who also smoked. No significant differences were found in the baseline characteristics between the intervention and control conditions (*p* > 0.05). We did find significance differences in three baseline characteristics between the two recruitment approaches. Compared to parents who were recruited via health care professionals, parents who were recruited via the mass media approach were significantly older (M = 41.83 years, SD = 7.07 vs. M = 37.72 years, SD = 6.91, t(81) = 2.58, *p* = 0.010), higher educated (46.7% vs. 13.2%, χ^2^ = 11.69, *p* = 0.003), and had significantly less often a child with chronic respiratory illness (10.0% vs. 66.0%, χ^2^ = 24.24, *p* < 0.001). 

### 3.2. Outcomes

#### 3.2.1. Smoking Cessation Rates

Table 2 shows the smoking cessation rates for the intervention and control conditions. The odds of reporting 7-day PPA at three months post-intervention was 7.54 higher for parents who received telephone counseling than for parents who received the self-help brochure (95% CI = 2.49–22.84). In addition, effects in favor of the telephone counseling were also found for 24 h PPA at three months post-intervention (OR = 6.64; 95% CI = 2.41–18.31) and 14-day PPA at four weeks after the designated quit date (OR = 8.23; 95% CI = 2.72–25.02). A sensitivity analysis on the primary outcome, in which parents who did not complete the follow-up assessment were considered quitters, revealed that the odds of reporting 7-day PPA at three months post-intervention was still 5.29 times higher in favor of the telephone counseling (95% CI = 2.06–13.55). No significant interaction effect was found between condition and the two recruitment approaches on the effectiveness of the telephone counseling (7-day PPA at three months post-intervention: OR = 6.08, 95% CI = 0.54–68.16; 24 h PPA at three months post-intervention: OR = 3.41, 95% CI = 0.37–31.07; 14-day PPA at four weeks after the designated quit date: OR = 1.23, 95% CI = 0.12–12.96). Yet, as presented in Table 3, relatively more parents who were recruited via the mass media approach quit smoking compared to parents who were recruited via the health care approach. Moreover, additional analyses showed some evidence that parents who were recruited via the mass media approach had higher odds to report 7-day PPA at three months post-intervention than parents who were recruited via the health care approach. However, adjusted logistic regression analyses showed no significant differences between the recruitment approaches and smoking cessation outcomes (Table 3). 

#### 3.2.2. Use of NRT and Smoking Cessation Medication

Significantly more parents in the intervention condition than parents in the control condition reported that they had used NRT during the study (66.7% vs. 26.3%, *χ*^2^ = 7.748, *p* = 0.005). No significant difference was found between the two conditions in using smoking cessation medication (11.1% vs. 10.5%, *χ*^2^ = 0.453, *p* = 0.501). 

#### 3.2.3. Secondary Outcomes among Parents Who did not Report Abstinence

Among the parents who did not report abstinence at three months post-intervention, no significant differences were found on the secondary outcomes (Table 4). Because only 14 parents indicated not having implemented a complete home smoking ban at the baseline assessment (see also Table 1), no analyses were performed to test whether significantly more parents in the intervention condition than parents in the control condition had implemented a complete home smoking ban during the study.

## 4. Discussion

This study provides insight into the effectiveness of a parent-tailored telephone smoking cessation counseling under more real-world conditions. As hypothesized, the odds of reporting 7-day PPA at three months post-intervention was 7.54 higher for parents who received telephone counseling than for parents who only received a self-help brochure on smoking cessation (95% CI = 2.49–22.84). These results yielded similar high effect sizes compared to the results of the efficacy trial at three months post-intervention (44.5% (telephone counseling) vs. 12.1% (self-help brochure), adjusted OR = 6.89, 95% CI = 4.18–11.36) [15]. A possible explanation for the small differences between 14-day PPA at 4-week after the designated quit date and 7-day and 24 h PPA at three months post-intervention could be the emphasis on relapse prevention in the telephone counseling. The last sessions with the counselors focus on dealing with craving and difficult moments. Having the opportunity to interact with a counselor on this topic and discuss personal situations that might make it harder to stay quit may decrease the likelihood that parents relapse at three months post-intervention. Results of other RCTs that examined the effectiveness of smoking cessation interventions tailored to parents at three months post-intervention were mixed. The majority of these studies showed non-significant results concerning smoking abstinence [11,13,20,31]. Only one study yielded positive effect sizes [18], which were lower than the effect sizes found in the efficacy trial [15] and the present study. The interventions that were provided to parents in the other studies included, among others, three telephone counseling sessions [11] or parents were offered to be referred to a quit line [20,31]. In two other studies, participating parents did not receive a telephone-based intervention but received, among others, an extensive (>10 min) anti-smoking message with a pediatric hospitalist and provision of NRT [13] or non-smoking mothers were, among others, motivated to advise their smoking partners to quit smoking [18]. Possibly, the combination of an intensive parent-tailored telephone counseling intervention (including 6 sessions), NRT and a parent-tailored self-help brochure is particularly beneficial. Further research should examine which factors of the intervention that were examined in the efficacy trial and the present study make the intervention so highly effective. In addition, to provide parents with effective smoking cessation interventions, more evidence is needed about whether the combination of elements (i.e., six telephone counseling sessions, NRT, and a parent-tailored self-help brochure) is indeed more effective than other smoking cessation interventions tailored to parents. 

One of the strengths of the efficacy trial was that the results revealed that the telephone counseling also yielded benefits to parents who had not quit smoking at three and 12 months post-intervention [15]. For example, significantly more parents who received the telephone counseling made more quit attempts by three and 12 months post-intervention than did parents in the control condition. In contrast to the efficacy trial, no significant differences were found among parents who did not report abstinence at three months post-intervention concerning the secondary outcomes increase in motivation to quit smoking, number of quit attempts during the study, and duration of longest quit attempt during the study. These unexpected findings could be explained by the small number of parents that were included in the analyses (*n* = 21 (intervention condition); *n* = 33 (control condition)). Although, these results were not significant, most results were directed in the expected way or in line with the efficacy trial.

The power calculation revealed that a sample size of at least 144 parents was needed in order to have sufficient power to examine the interaction effect between condition and recruitment approach with a large effect. Because only 83 parents were included in the analyses, this study was underpowered to examine the interaction effect. As our sample size did not allow us to interpret potential interaction effects, we concentrated on alternative ways to obtain information about differences in the effectiveness of the telephone counselling between the two approaches. Descriptive results illustrated that relatively more parents who were recruited via the mass media approach quit smoking compared to parents who were recruited via the health care approach. In addition, we found some evidence that parents who were recruited via the mass media approach had higher odds to report 7-day PPA at three months post-intervention than parents who were recruited via the health care approach. These results point in the direction of a mass media approach being a more effective recruitment approach than the health care approach. However, caution is warranted as - because of the small sample size - our findings may be less reliable (reflected in the broad confidence intervals in the regression analyses) and are not very robust. A larger sample size is needed to examine whether the effectiveness of the telephone counseling depends on the recruitment approach. For implementation purposes it is not sufficient to solely look at the impact of a recruitment approach on the effectiveness of the intervention. It is also important to consider the success rate of the possible recruitment approaches [32]. In other words, it is necessary to consider which recruitment approach is most successful in recruiting the target group (i.e., how many people were recruited and how many of them actually started the intervention). In brief, to determine which recruitment approach should be employed in recruiting parents for telephone counseling, further research should also examine the success rates of the health care and mass media approaches. In addition, the present study showed that it was challenging to recruit smoking parents, as the number of parents that was needed was not reached due to a high drop-out of parents after they had been referred. This stresses the need for more information on why parents decided not to be willing to receive telephone counseling or to participate in the RCT and how recruitment could be improved. 

### 4.1. Strengths and Limitations

A major strength of the present study is that the telephone counseling was tested in an effectiveness trial under more real-world conditions (i.e., parents were recruited via approaches that could be used as recruitment approaches when the telephone counseling is implemented in the future, parents did not receive 100 euros for participating in the study, and parents had to pay for the costs of the telephone counseling if it was not covered by their health insurance). As the distinction between efficacy and effectiveness should be viewed as a spectrum instead of a strict dichotomy [33], we think that, based on the characteristics of the present study, this study more closely resembles an effectiveness than an efficacy trial. A second strength is that parents were recruited via two recruitment approaches that were set up in everyday practice, without any interference to stimulate the inclusion. Both of these strengths contributes to the generalizability of our findings to everyday practice. A third strength is that Russell Standard (Clinical) version 2 [29] was used to assess smoking abstinence which enables meaningful comparisons between different studies.

In addition to its strengths, this study has four major limitations. First, in contrast to the efficacy trial, the effectiveness of the telephone counseling was not examined at the long term (e.g., 12-month follow-up). In the course of the study, the decision was made to extend the recruitment of parents in order to increase the inclusion. As a result, a 12-month follow-up was no longer possible within the course of the study. The results of the efficacy trial revealed that the telephone counseling remained effective at 12 months post-intervention (34.0% of the parents in the intervention condition reported 7-day PPA vs. 18.0% of the parents in the control condition; OR = 2.81, 95% CI = 1.76–4.49) [15]. Based on these results and the findings of the present study, we expect that the telephone counseling remains effective in the long term. The second limitation is that the self-reported abstinence status was only biochemically validated for a few parents. A subsample of parents who reported smoking abstinence at three months follow-up was approached for biochemical validation (*n* = 17). Participation required some saliva to be collected from parents during a home visit. The majority of the approached parents (*n* = 13) did not want to participate for various reasons. Results of the biochemical validation confirmed the quit status of two parents. With respect to the other two parents who participated in the biochemical validation, results showed that they were either heavily exposed to SHS or were light smokers. Because biochemical validation confirmed the quit status of the majority of parents (81.8%) in the efficacy trial [15], we do not expect this confirmation rate to be lower for the present study. A third limitation is that the 95% CIs of the effect sizes concerning the smoking cessation outcomes were quite wide. Therefore, caution is needed in interpreting these results. Finally, it should be recognized that the number of parents in this study is relatively low, given that several channels were involved to recruit smoking parents. Because only a small proportion of the source population was included in our study, generalizability of our conclusions to the larger population of smoking parents is limited to some extent.

### 4.2. Implications for Practice and Suggestions for Further Research

This effectiveness trial showed that telephone smoking cessation counseling tailored to parents is effective in helping parents to quit smoking within three months post-intervention, when tested under more real-world conditions. However, it remains unclear whether telephone counseling is also cost-effective. Information on the cost-effectiveness of a health promotion intervention is essential for policy makers to decide whether or not to implement the intervention. Therefore, further research should examine the cost-effectiveness of the parent-tailored telephone smoking cessation counseling. In addition, intervention success is not solely determined by the effectiveness of the intervention, but is also affected by the extent to which people have access to and are willing to use the intervention [32]. As mentioned previously, for the implementation of the telephone counseling, it is crucial to test which recruitment approach is most successful in recruiting parents and how recruitment could be improved. When considering the success rates of the recruitment approaches, it is also important to assess their costs of the recruitment approaches. If recruitment approaches result in high recruitment costs, policy makers may be less likely to employ those approaches to implement the interventions. Finally, it is important to explore how parents who received the telephone counseling experienced the intervention and whether any improvements can be made in order to expand the benefits of the telephone counseling. 

## 5. Conclusions

The results of the present effectiveness study show that a parent-tailored telephone smoking cessation counseling intervention is effective in encouraging parents to quit smoking within three months. Implementation of the telephone counseling could be considered, taking the difficulties of the recruitment of parents into account. To increase the impact of this evidence-based intervention in terms of public health and tobacco control among parents and their children, it is essential to gain insight into how recruitment of parents via different recruitment approaches (i.e., health care vs. mass media) could be improved. In addition, more insight is needed about the cost-effectiveness of the telephone counseling.

## Figures and Tables

**Table 1:** Key characteristics of parents at baseline.

Characteristics		Total Sample (*N* = 83)	Telephone Counseling (*n* = 45)	Self-Help Brochure (*n* = 38)
Age (mean, SD)		39.2 (7.20)	39.3 (7.66)	39.1 (6.71)
Gender % (*n*)				
	Male	42.2 (35)	46.7 (21)	36.8 (14)
Nationality % (*n*)				
	Dutch	94.0 (78)	93.3 (42)	94.7 (36)
Educational level % (*n*)				
	Low	27.7 (23)	28.9 (13)	26.3 (10)
	Medium	47.0 (39)	46.7 (21)	47.4 (18)
	High	25.3 (21)	24.4 (11)	26.3 (10)
Marital status % (*n*)				
	Married/living together	71.1 (59)	73.3 (33)	68.4 (26)
	Divorced/widowed and now single	7.2 (6)	4.4 (2)	10.5 (4)
	Single	16.9 (14)	17.8 (8)	15.8 (6)
Employment status % (*n*)				
	Unemployed	18.1 (15)	20.0 (9)	15.8 (6)
	Homemaker	7.2 (6)	6.7 (3)	7.9 (3)
	Paid employment	74.7 (62)	73.3 (33)	76.3 (29)
Medical treatment % (*n*)		15.7 (13)	15.6 (7)	15.8 (6)
Cardiovascular disease % (*n*)		7.2 (6)	11.1 (5)	2.6 (1)
Chronic respiratory illness % (*n*)		12.0 (10)	13.3 (6)	10.5 (4)
Chronic respiratory illness child % (*n*)		45.8 (38)	40.0 (18)	52.6 (20)
Cigarettes per day (mean, SD)		15.5 (6.67)	15.0 (5.48)	16.0 (7.88)
Years of smoking (mean, SD)		20.4 (7.39)	21.1 (8.0)	19.6 (6.66)
FTND score (mean, SD)		4.3 (2.29)	4.4 (2.19)	4.3 (2.45)
Complete home smoking ban % (*n*)		83.1 (69)	86.7 (39)	78.9 (30)
Partner smoking % (*n*)				
	Yes	34.9 (29)	33.3 (15)	36.8 (14)

Notes. No significant differences (*p* > 0.05) were found in any measure between the intervention and control conditions.

**Table 2:** Smoking cessation outcomes between intervention (*n* = 45) and control condition (*n* = 38).

Smoking Cessation Outcomes	Telephone Counseling % (*n*)	Self-Help Brochure % (*n*)	OR (95% CI) *
Primary outcome			
7-day PPA at 3-month FU	53.3 (24)	13.2 (5)	7.54 (2.49–22.84)
Secondary outcomes			
24 h PPA at 3-month FU	60.0 (27)	18.4 (7)	6.64 (2.41–18.31)
14-day PPA at 4-week after the designated quit date	55.6 (25)	13.2 (5)	8.23 (2.72–25.02)

Notes. Intention-to-treat analyses were carried out. In accordance with the Russell Standard criteria, parents who did not complete the follow-up assessment were considered “smokers”. CI = confidence interval. PPA = point prevalence abstinence. FU = follow-up * Because none of the baseline variables shown in Table 1 were significantly correlated with the primary outcome, no adjusted ORs were calculated.

**Table 3:** Smoking cessation outcomes between condition and recruitment approaches.

Smoking Cessation Outcomes		Telephone Counseling % (*n*)		Self-Help Brochure % (*n*)		OR (95% CI)	Adjusted OR (95% CI) ^1^
		Health Care (*n* = 29)	Mass Media (*n* = 16)	Health Care (*n* = 24)	Mass Media (*n* = 14)		
Primary outcome							
	7-day PPA at 3-month FU	37.9 (11)	81.3 (13)	12.5 (3)	14.3 (2)	2.79 (1.09–7.14)	2.90 (0.88–9.56)
Secondary outcomes							
	24 h PPA at 3-month FU	48.3 (14)	81.3 (13)	16.7 (4)	21.4 (3)	2.22 (0.89 –5.55)	1.63 (0.52–5.04)
	14-day PPA at 4-week after the designated quit date	44.8 (13)	75.0 (12)	8.3 (2)	21.4 (3)	2.53 (0.997–6.44)	1.91 (0.60–6.03)

Notes. Intention-to-treat analyses were carried out. In accordance with the Russell Standard criteria, parents who did not complete the follow-up assessment were considered “smokers”. ^1^ Adjusted for age, educational level, and chronic respiratory illness child.

**Table 4:** Secondary outcomes among parents in the intervention (*n* = 21) and control condition. (*n* = 33) who did not report abstinence at three months post-intervention.

Secondary Outcomes		Telephone Counseling	Self-Help Brochure	*p*
Increase in motivation to quit during the study (M, SE)		−1.17 (0.76)	−1.00 (0.42)	>0.05
Number of quit attempts during the study (M, SE)		2.2 (0.37)	1.7 (0.21)	>0.05
Duration of longest quit attempt during the study *				>0.05
	Less than one week % (*n*)	25.0 (4)	48.3 (14)	
	One week or more % (*n*)	75.0 (12)	51.7 (15)	

Notes. Intention-to-treat analyses were carried out. In accordance with the Russell Standard criteria, parents who did not complete the follow-up assessment were considered “smokers”. Because only 14 parents indicated not having implemented a complete home smoking ban at the baseline assessment, no analyses were performed to test whether significantly more parents in the intervention condition than parents in the control condition had implemented a complete home smoking ban during the study. * Duration of longest quit attempt was calculated among smokers who reported a quit attempt (intervention condition: *n* = 16; control condition: *n* = 29).
